# Recent advances in chemiluminescence probes for Tumor microenvironment with applications in cancer diagnosis and therapy

**DOI:** 10.1016/j.pscia.2026.100113

**Published:** 2026-02-16

**Authors:** Dongnan Guo, Xiaofang Hou, Sicen Wang

**Affiliations:** a School of Pharmacy, Xi'an Jiaotong University, 76 Yanta West Road, Xi'an, 710061, Shaanxi, China; b Shaanxi Engineering Research Center of Cardiovascular Drugs Screening and Analysis, Xi'an, 710061, Shaanxi, China

**Keywords:** Tumor microenvironment, Chemiluminescent probes, Cancer diagnosis and therapy, Dioxetane, Luminol, Peroxalate

## Abstract

Optical imaging enables noninvasive visualization of molecular processes in living systems and plays an essential role in cancer research. Among available optical modalities, chemiluminescence imaging (CLI) offers a distinctive advantage for probing the tumor microenvironment (TME) by eliminating external excitation and minimizing background interference. Because photon emission arises directly from chemical reactions, CLI converts endogenous biochemical activity within the TME into localized and selective optical signals.

This review examines recent advances in the design of TME-responsive chemiluminescence (CL) probes for cancer diagnosis and therapy. Despite rapid methodological progress, a persistent knowledge gap remains in quantitatively correlating molecular design with heterogeneous TME characteristics, while simultaneously addressing translational constraints such as tissue penetration, pharmacokinetics, and probe manufacturability. The historical evolution of three major CL scaffolds—luminol, peroxyoxalate, and dioxetane—is first outlined to illustrate how structural innovations have progressively improved biological compatibility and functional specificity. Design strategies targeting representative TME hallmarks, including redox imbalance, hypoxia, acidic pH, and aberrant enzyme activity, are then systematically discussed. By comparing applications across distinct tumor models, including hepatocellular, breast, and lung cancers, this review highlights how tumor-type-specific biochemical heterogeneity fundamentally shapes imaging performance and therapeutic relevance. The review concludes with a perspective on remaining challenges and emerging directions, emphasizing standardization, multifunctional integration, and application-driven probe design to advance CLI toward broader biomedical and translational impact.

## 1 Introduction

Over the past decade, optical molecular imaging has become an essential tool in cancer research by enabling sensitive and dynamic visualization of disease-associated processes in living systems [1,2]. Compared with macroscopic imaging modalities such as magnetic resonance imaging (MRI), computed tomography (CT) and positron emission tomography (PET), optical imaging offer superior molecular sensitivity and temporal resolution at the preclinical scale. Within this broad landscape, chemiluminescence imaging (CLI) has attracted increasing attention as a complementary modality to fluorescence-based techniques, particularly in scenarios where external excitation is limited by autofluorescence, light scattering, and photobleaching [[3], [4], [5], [6], [7]]. Because CL photons are generated through chemical reactions rather than external excitation [8], CLI intrinsically features low background and high signal-to-noise ratios in optically complex biological environments; mechanistic advances in organic chemiexcitation have further supported more rational probe optimization in aqueous media.

The growing interest in CLI is closely linked to an improved understanding of cancer as a disease governed not only by malignant cells but also by the tumor microenvironment (TME) [9]. The TME is now recognized as a dynamic ecosystem shaped by tumor–stroma interactions and organ-specific “seed-and-soil” effects [[10], [11], [12], [13]], which critically influence tumor progression, metastasis, and therapeutic response. Accordingly, imaging strategies capable of reporting on microenvironmental chemistry and function have gained increasing importance in both mechanistic cancer studies and translational research.

Chemiluminescence (CL) is particularly well suited for interrogating the TME because photon emission can be coupled to endogenous biochemical events within tumors rather than relying on external excitation. Consistent with this principle, activatable fluorescence and bio/chemiluminescence probe designs have been developed to translate tumor-associated signals—including redox imbalance, enzyme activity, and hypoxia-linked processes—into localized optical readouts. In parallel, shifting emission toward the near-infrared (NIR) region remains important for improving in vivo imaging depth, given the well-established penetration benefits at longer wavelengths. Recent work has therefore emphasized NIR and even NIR-II CL designs and platforms for in vivo imaging and therapeutic integration [[14], [15], [16], [17], [18], [19], [20], [21], [22]].

From a molecular-design perspective, contemporary CLI systems are commonly discussed in terms of three major CL scaffold families: luminol, peroxyoxalate, and 1,2-dioxetanes. Luminol chemistry has a long history in analytical and biomedical contexts and has been extensively used to report oxidative processes, including in vivo applications and methodological studies [[23], [24], [25], [26], [27], [28]]. Peroxyoxalate chemiluminescence, originally established as a high-efficiency chemiexcitation system, provides a foundation for energy-transfer designs and has been adapted for in vivo peroxide imaging in nanoparticle formats [[29], [30], [31], [32]]. Meanwhile, 1,2-dioxetanes introduced chemically and enzymatically triggerable activation paradigms and, more recently, have undergone rapid innovation in modular synthesis, wavelength tuning (including NIR emission), and sensitivity enhancement [[33], [34], [35], [36], [37], [38], [39], [40], [41]].

Despite substantial progress, several challenges continue to shape the field. A persistent trade-off remains between achieving deeper tissue imaging through red-shifted/NIR emission and maintaining high photon output and robustness under physiological conditions. Moreover, increasing architectural complexity raises practical questions regarding standardization, manufacturability, and translational evaluation. As a result, while the literature contains numerous individual probe designs, unified frameworks that connect molecular architecture with quantitative TME characteristics and in vivo performance remain comparatively limited.

Existing reviews have summarized recent advances in CLI and dioxetane-based probe development. However, a synthesis that explicitly integrates scaffold evolution, activation chemistry, tumor-type heterogeneity, and translational considerations is still needed. To address this gap, this review is structured as follows: Section 2 outlines the historical evolution of the major CL scaffolds (luminol, peroxyoxalate, and dioxetanes) through a generational perspective. Section 3 systematically analyzes the design strategies for TME-responsive CL probes, focusing on key biochemical triggers such as redox imbalance, gasotransmitters, and enzymatic/hypoxic pathways. Section 4 examines the performance and representative applications of these probes across distinct tumor models (e.g., hepatocellular, breast, and lung cancers), highlighting how tumor-type-specific biochemical heterogeneity shapes imaging outcomes and therapeutic potential. Methodological discussions in tumor-type specificity and practical constraints of CL probe design are then presented in Section 5. Finally, Section 6 concludes with a critical discussion of remaining challenges and future perspectives, emphasizing the need for standardization, multimodal integration, and application-driven design to advance CLI toward broader biomedical impact.

## 2 Historical evolution of CL for biomedical Imaging:A generational perspective

The evolution of CL imaging for biomedical applications can be viewed as a progression through distinct generational stages defined by changes in chemical scaffolds, activation control, and biological deployability. A generational framework helps clarify how successive design strategies addressed specific limitations while introducing new constraints that continue to influence current research directions.

### 2.1 First-generation systems: foundational chemistry and intrinsic limitations

First-generation CL systems established the feasibility of chemical light generation in biological contexts and were dominated by luminol- and peroxyoxalate-based chemistries. Luminol-dependent CL became widely used for reporting oxidative processes, with extensive clinical and non-clinical applications summarized in later overviews [23,42]. Early methodological studies also established luminol chemiluminescence as a practical readout for immune and phagocytic functions [[25], [26], [27]]. Subsequent luminol-derived probes, such as L-012, enabled in vivo imaging of reactive oxygen and nitrogen species, while later reevaluations clarified interpretational considerations for superoxide detection [24,28].

Peroxyoxalate chemiluminescence introduced a distinct chemiexcitation logic, in which chemical energy generated by oxalate–peroxide reactions can excite fluorescent acceptors, enabling tunable emission profiles [29]. Recent mechanistic analyses have further clarified chemiexcitation processes and stability considerations of peroxyoxalate systems in aqueous media, providing an updated foundation for rational probe optimization. The incorporation of peroxyoxalate reactions into nanoparticle platforms subsequently demonstrated the feasibility of in vivo hydrogen peroxide imaging and highlighted the potential of carrier-assisted CL systems [[30], [31], [32],^43^].

Early work on 1,2-dioxetanes also belongs to this foundational stage, particularly studies demonstrating chemical and enzymatic triggering mechanisms suitable for bioassays [44]. While these systems introduced the concept of masked CL activation, broader application in complex biological environments required subsequent advances in brightness, modularity, and aqueous compatibility.

### 2.2 Second-generation systems: programmable and trigger-responsive dioxetanes

Second-generation CLI emerged with the rise of trigger-responsive dioxetane luminophores and a design philosophy centered on molecular programmability [[33], [34], [35], [36], [37], [38], [39]]**.** In these chemical systems, CL emission remains silent until a specific biochemical trigger unmasks the emissive pathway, enabling improved spatial and temporal control over signal generation and advanced biochemical specificity.

**Fig. 1:**
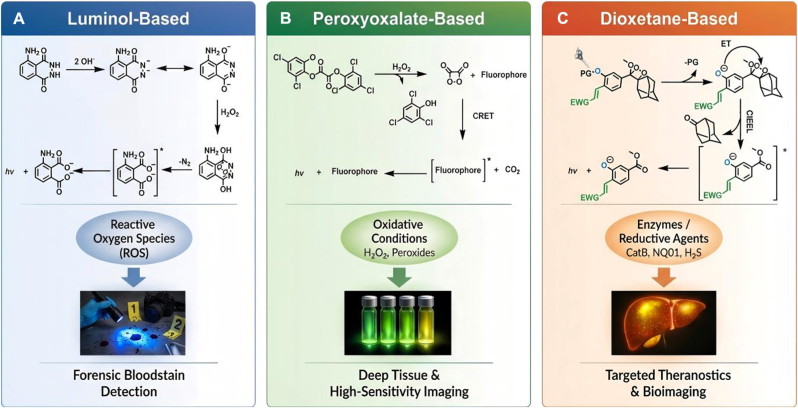
**Three Major CL Scaffolds and Representative Biomedical Imaging Applications.** Luminol-based CL system (left) undergoes peroxidase-catalyzed oxidation to yield an excited 3-aminophthalate dicarboxylate, primarily reporting oxidative bursts. Peroxyoxalate-based CL system (middle) relies on a bimolecular reaction with hydrogen peroxide to form a high-energy dioxetanone intermediate, which transfers energy to a nearby fluorophore via the chemically initiated electron exchange luminescence (CIEEL) mechanism. Dioxetane-based CL platforms feature a "turn-on" design logic: a spiro-adamantane moiety confers thermal stability, while emission is gated by a specific masking group. Upon cleavage by a target biomarker—such as the dephosphorylation by alkaline phosphatase (ALP) or cleavage by specific proteases—the unstable phenolate anion is generated, initiating decomposition and light emission. This structural shift allows for the precise interrogation of non-oxidative enzymatic targets within the TME.

During this stage, modular and convergent synthetic strategies enabled rapid access to diversified dioxetane luminophores, while structural tuning allowed emission wavelengths to be adjusted and signal properties optimized [33,40]. Sensitivity-enhancing design innovations, including strain-accelerated chemiexcitation, further pushed detection performance in bioassay contexts and imaging applications [3,45]. Color modulation approaches also supported multiplexed CL readouts using phenoxy-1,2-dioxetane luminophores [35]. The structural characteristics and activation mechanisms of the three major CL scaffolds discussed above are summarized in Fig. 1.

### 2.3 Current stage: NIR/NIR-II expansion, functional integration, and translational emphasis

The current generation of CLI is defined by a multi-faceted expansion beyond foundational probe development, driven by an imperative for clinical translation and deeper biological interrogation. This stage is marked by four major interconnected frontiers:

1. **Spectral expansion into NIR/NIR-II and beyond.** A primary focus is the extension of emission wavelengths into the near-infrared I/II (NIR-II, 1000–1700 nm) and even longer windows to drastically improve tissue penetration depth and spatial resolution in vivo. Moving beyond simple emission red-shifting, current innovations include direct-activated NIR CL platforms that circumvent energy-transfer losses, and small-molecule NIR probes for sensitive imaging of specific biomarkers like H_2_S [14,46]. Research also explores emissive materials with ultra-narrowband emission or prolonged afterglow in these spectral regions to further enhance signal-to-background ratios [[47], [48], [49], [50]].
2. **Advanced functional integration and theranostics.** The field is rapidly evolving from passive imaging to active intervention. Current work emphasizes CL-driven theranostics, where the emitted light not only reports but also initiates therapeutic action [[51], [52], [53]].
3. **Engineering for biological complexity and specificity.** Recognizing the heterogeneity of the TME, current probe design prioritizes high-fidelity biochemical reporting [19,33,51].
4. **Translational-oriented system engineering.** A significant shift is underway from proof-of-concept molecules to engineered systems designed with clinical application constraints in mind [48,54].

**In summary,** the current generation represents a maturation phase where CL is being refined as a robust, multifunctional, and translationally relevant toolkit. The convergence of advanced optical engineering, sophisticated molecular design, and therapeutic integration is positioning CLI not merely as an alternative imaging modality, but as a unique platform for precise cancer diagnosis, mechanistic study of the TME, and image-guided intervention. This evolution has been fundamentally driven by the sequential development and refinement of three core CL scaffolds. To understand how each core's intrinsic properties dictate its suitability for interrogating specific TME features, their defining characteristics, historical roles, and design trade-offs are strategically mapped in the following.

This table underscores that CLI performance is governed primarily by the compatibility between probe activation chemistry and the biochemical heterogeneity of the TME, rather than by photon output alone. On this basis, Section 3 systematically reviews TME-responsive CL probes according to their underlying biochemical activation mechanisms.

## 3 Biochemical activation strategies for TME-responsive CL probes

Guided by the scaffold–hallmark matching summarized in Table 1, contemporary CL probe design has increasingly converged on a central principle: effective imaging of the TME requires the translation of specific biochemical activities into controlled chemiexcitation events [55,56]. Rather than targeting tumor cells directly, TME-responsive CL probes exploit dysregulated chemical processes—such as redox imbalance, aberrant gasotransmitter production, and enzyme activity—to generate localized optical signals with minimal background interference [57].

**Table 1:** Strategic mapping of CL scaffolds to TME hallmarks.

CL Scaffold (Generation)	Typical λ_em (nm)	Strengths	Dominant TME Hallmark	Representative Biomarker (tumor range)	Activation Chemistry	Key Design Constraint	References
**Luminol** (1st Gen, foundational reporter)	420–460	Simple, catalytic amplification	Oxidative burst/inflammation	H_2_O_2_ (≈10–100 μM) O_2_•^-^ (transient)	Peroxidase-catalyzed oxidation	Severe tissue attenuation Poor biochemical specificity	[[23], [24], [25], [26], [27], [28],^58^,^59^]
**Peroxyoxalate** (1st–2nd Gen, energy-transfer system)	450–650	Tunable color via dye; high yield	Oxidative stress	H_2_O_2_ (locally high)	Oxalate–H_2_O_2_ reaction + CRET to fluorophore	Requires high local peroxide Hydrolytic instability	[[29], [30], [31], [32]]
**Dioxetane** (2nd–3rd Gen, programmable platform)	500–1200 (with π-extension)	Triggerable, biocompatible, NIR-extendable	Redox imbalance	H_2_O_2_, ONOO^−^, O_2_•^-^	Boronate/phosphonate oxidation	Brightness–red-shift trade-off	[16,45,[60], [61], [62], [63], [64], [65], [66], [67], [68], [69], [70], [71]]
			Thiol/sulfur metabolism	H_2_S (≈10–100 μM) GSH (mM, intracellular)	Azide reduction Disulfide cleavage	Competing thiols; spatial heterogeneity	[14,46,[72], [73], [74]]
			Hypoxia-linked enzymatic activity	NTR (↑ under hypoxia)	Nitro reduction/self-immolation	Enzyme-expression variability	[7,57,75]
			Enzyme dysregulation	ALP, APN, FAP, ENPP-1, CatB, NQO1	Enzyme-dependent (↑2–50 × )	NIR-favored High S/N	[[76], [77], [78], [79], [80], [81], [82], [83], [84]]
			Acidic metabolism	Extracellular pH 6.5–6.9	Acid-labile linkers	Modest pH contrast	[51]
			Multimodal/theranostic coupling	ROS/enzymes + therapy	Dual or AND-gated logic	System complexity	[18,34,51,52,[85], [86], [87]]

**Fig. 2:**
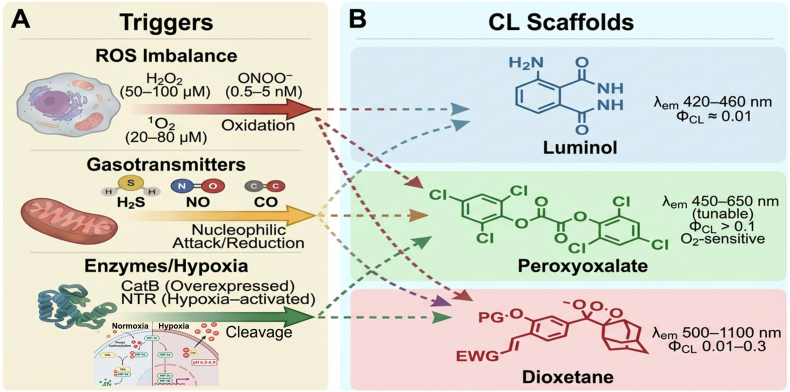
CL meets TME.

A distinctive advantage of CL imaging lies in its excitation-free signal generation. Because photon emission arises directly from chemical reactions, CL probes can report low-abundance, spatially heterogeneous, or transient biochemical signals that are difficult to access using excitation-dependent modalities [48]. In this section, major classes of TME-responsive CL probes are discussed according to their underlying biochemical activation mechanisms, with an emphasis on how molecular design choices shape specificity, sensitivity, and in vivo performance (Fig. 2).

### 3.1 Redox imbalance as a trigger for CL activation

Redox dysregulation is a pervasive feature of the TME, driven by altered mitochondrial metabolism, oncogenic signaling, immune infiltration, and inflammatory processes. Elevated levels of reactive oxygen species (ROS) [88] and reactive nitrogen species (RNS), including hydrogen peroxide, superoxide, hypochlorite, and peroxynitrite, have therefore been widely exploited as endogenous triggers for CL probe activation [58,89].

Early CL-based approaches established the feasibility of visualizing oxidative stress in vivo, demonstrating that chemiexcitation could serve as a direct reporter of redox chemistry without external illumination [31]. Subsequent molecular refinements improved detection sensitivity, reaction selectivity, and spatial localization, enabling more precise interrogation of oxidative processes within tumor tissues [60,90]. Importantly, the intrinsic coupling between redox reactivity and photon generation allows CL to reflect local chemical activity rather than accumulated probe concentration alone [57].

Beyond hydrogen peroxide, probes responsive to specific ROS and RNS have been developed to capture immune-associated oxidative bursts and inflammatory signaling within tumors [16,45,[62], [63], [64], [65], [66], [67]]. These studies collectively illustrate how redox-responsive CL can be tuned to distinct biochemical contexts through appropriate choice of activation chemistry and luminophore scaffold, while also highlighting inherent trade-offs between trigger abundance, signal duration, and tissue penetration.

### 3.2 Gasotransmitter-responsive CL probes

In addition to classical redox species, gaseous signaling molecules such as nitric oxide, carbon monoxide, and hydrogen sulfide play increasingly recognized roles in tumor metabolism, angiogenesis, and immune regulation [91]. Among these gasotransmitters, hydrogen sulfide has attracted particular attention due to its elevated production in several tumor types and its involvement in redox homeostasis and mitochondrial function [[92], [93], [94]].

CL probes responsive to hydrogen sulfide typically use its nucleophilicity or reducing capability to initiate chemiexcitation. Early proof-of-concept designs demonstrated selective H_2_S-triggered light emission in biological systems, establishing the feasibility of gasotransmitter imaging using CL [95]. More recent efforts have focused on enhancing in vivo performance by improving activation specificity and extending emission into the near-infrared region to facilitate deep-tissue imaging [20,33,85].

Compared with fluorescence-based approaches, CL gasotransmitter probes offer improved signal-to-background ratios and reduced susceptibility to tissue autofluorescence. These properties are particularly advantageous in the TME, where gasotransmitter concentrations can be spatially heterogeneous and temporally dynamic. Nevertheless, competing thiols and variable endogenous levels impose important constraints that must be addressed through careful molecular design.

### 3.3 Enzyme- and hypoxia-responsive CL systems

Enzymatic dysregulation represents another defining biochemical characteristic of the TME. Adjusted expression and activity of enzymes involved in metabolism, extracellular matrix remodeling, and immune modulation provide opportunities for activatable CL probe design. Enzyme-responsive systems typically rely on substrate cleavage or structural unmasking to initiate light emission, thereby directly coupling enzymatic activity to optical output [[76], [77], [78], [79], [80], [81], [82], [83], [84]].

Hypoxia, arising from abnormal tumor vasculature and rapid cellular proliferation, constitutes a complementary and clinically relevant TME feature [96]. Hypoxia-responsive CL probes target reductive enzymes or oxygen-dependent reaction pathways to visualize regions associated with aggressive tumor phenotypes and therapeutic resistance [7,57,75]. Together, enzyme- and hypoxia-responsive designs extend CL beyond redox chemistry to broader functional dimensions of tumor biology.

### 3.4 Toward CL-enabled theranostic strategies

As CL probe architectures have matured, increasing attention has been directed toward integrating diagnostic imaging with therapeutic function. In such systems, CL reactions not only report on tumor-associated biochemical activity but also initiate downstream therapeutic processes, such as photodynamic therapy or prodrug activation [18,34,51,52,[85], [86], [87]].

A schematic overview of biochemical activation strategies enabling TME-responsive CL probes to translate dysregulated biochemical hallmarks into excitation-free optical signals.

In most reported examples, theranostic capability emerges as a consequence of rational probe design rather than as an isolated objective. By harnessing endogenous tumor chemistry to drive both signal generation and therapeutic action, CL-based systems offer a pathway toward spatially confined intervention with intrinsic optical feedback. This evolution underscores a broader shift in the field, positioning CL as a functional transduction modality rather than a purely diagnostic tool.

## 4 Design principles, failure modes, and contextual constraints of TME-responsive CL probes

A recurring misconception in the development of CL probes is that improving molecular reactivity or lowering activation thresholds will necessarily translate into superior in vivo imaging performance. Accumulating evidence across tumor models indicates that this assumption is frequently invalid [6,45]. In living systems, CL signal generation is governed by a multilayered interplay among activation chemistry, probe delivery, and tumor-specific biochemical permissiveness. Consequently, performance limitations more often arise from contextual mismatches than from intrinsic deficiencies in chemiexcitation mechanisms alone.

Importantly, these constraints do not appear sporadically but instead manifest in a tumor-type–dependent and reproducible manner. As summarized in Table 2 (more detailed information is contained in SI Table S1), different cancers are characterized by distinct dominant TME features—such as redox imbalance, sulfur metabolism, enzyme dysregulation, or hypoxia—which collectively define the effective operating window for specific CL scaffolds and activation triggers. From this perspective, tumor models should be regarded not merely as disease categories, but as biochemically defined evaluation environments that fundamentally shape probe activation efficiency, signal stability, and interpretability.

**Table 2:** CLI applications in major tumor types.

Targeted TME feature	CL scaffold	Representative tumor model	Emission region	Signal duration	Key design insight	References
Oxidative stress	Dioxetane	HCC (orthotopic/xenograft)	Visible–NIR	Minutes–hours	Robust but model-dependent	[62,55,97]
Sulfur metabolism (H_2_S)	Dioxetane/BAC	Breast/HCC	Visible–NIR	Hours	Specific but thiol-competitive	[33]
Enzyme dysregulation (ALP, CatB, NQO1)	Dioxetane	Breast/HCC	Visible–NIR	Minutes	Accessibility-limited	[81,82,84,97]
Hypoxia-associated enzymes (NTR)	Dioxetane	Lung tumors/Breast	Visible	Short	Perfusion-limited	[52,65]
Inflammation (MPO–ROS cascade)	Luminol-based	Inflammation-rich tumors	Blue–Visible	Minutes	Bright but shallow	[32,98,99]
Multimodal/theranostic coupling	Dioxetane/hybrid	HCC/breast tumors	Visible–NIR	Variable	Functionality–efficiency trade-off	[60,61,90]

### 4.1 TME as a determinant of CL activation landscapes

Tumors differ markedly in redox status, enzyme expression profiles, oxygen availability, vascular permeability, and stromal organization. These parameters jointly determine whether endogenous chemiexcitation reactions can proceed efficiently and whether the generated photons can be detected with sufficient signal-to-noise ratios in vivo. Consequently, probe architectures that perform robustly in one tumor model often exhibit reduced reliability or inconsistent behavior when directly applied to other tumor types.

As outlined in Table 2, hepatocellular carcinoma (HCC) represents a tumor context with a highly permissive biochemical landscape for CL activation, owing to its pronounced redox imbalance, active sulfur metabolism, and enzyme-rich microenvironment [48,62]. By contrast, breast, colorectal, and lung tumors typically present more heterogeneous or restrictive biochemical conditions, imposing stricter constraints on activation efficiency and signal propagation.

### 4.2 Redox-responsive probe designs: context-enabled robustness rather than universal validity

**Fig. 3:**
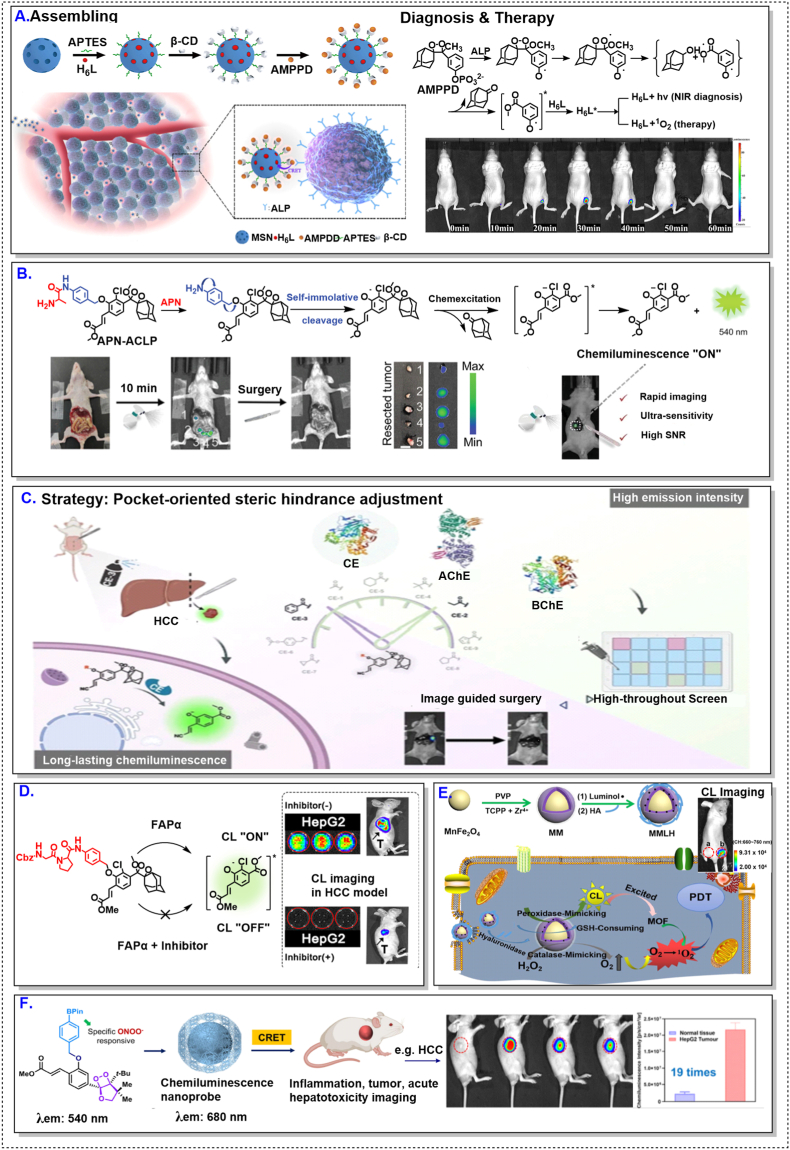
CL probe performance in hepatocellular carcinoma models. Representative enzyme- and redox-responsive CL probes evaluated in hepatocellular carcinoma (HCC) models. Across diverse activation chemistries, HCC consistently supports strong and sustained CL outputs. These examples illustrate the high biochemical permissiveness of the liver-TME toward CL activation. All above figures were reproduced with proper copyright obtained.

Redox-responsive CL probes dominate the current literature, reflecting the central role of oxidative stress in cancer biology. As illustrated in Fig. 3, HCC models have consistently yielded strong and reproducible CL signals across a wide range of redox-activated probe architectures [62,55,97,100]. However, as indicated by the comparative landscape in Table 2, this apparent robustness should be interpreted primarily because of the liver TME rather than as evidence of universally effective probe design.

The HCC microenvironment is characterized by elevated basal ROS levels, active sulfur metabolism, and abundant redox-active enzymes, collectively lowering the activation threshold required for CL signal generation [79,80]. As a result, structurally diverse probes—some of which would be considered suboptimal in less permissive contexts—can nonetheless produce robust signals in HCC models.

When similar redox-responsive probes are applied to breast, colorectal, or lung tumors, performance frequently deteriorates [97,98,100]. In these settings, heterogeneous redox landscapes, variable enzyme expression, and spatially restricted probe penetration introduce pronounced signal variability. Increasing trigger sensitivity under such conditions often amplifies background activation rather than contrast. These observations underscore a critical limitation: redox responsiveness constitutes a context-dependent advantage, not a transferable design rule.

### 4.3 Enzyme-activated CL probes: specificity constrained by accessibility

Enzyme-activated CL probes aim to improve molecular specificity by exploiting dysregulated enzyme expression in tumors, such as cathepsins or oxidoreductases. As summarized in Table 2 and Table S1, these strategies are frequently applied to tumor types where enzyme dysregulation is a dominant microenvironmental feature, including breast cancer [33,81,82,101].

In practice, however, enzyme responsiveness is constrained less by catalytic efficiency than by biological accessibility. Heterogeneous enzyme distribution, limited probe delivery, and basal enzyme expression in non-malignant or inflamed tissues collectively restrict spatial specificity and increase the risk of off-target CL activation [[102], [103], [104]]. This highlights an important distinction that is often underappreciated: enzyme specificity at the molecular level does not guarantee spatial specificity at the tissue level.

**Fig. 4:**
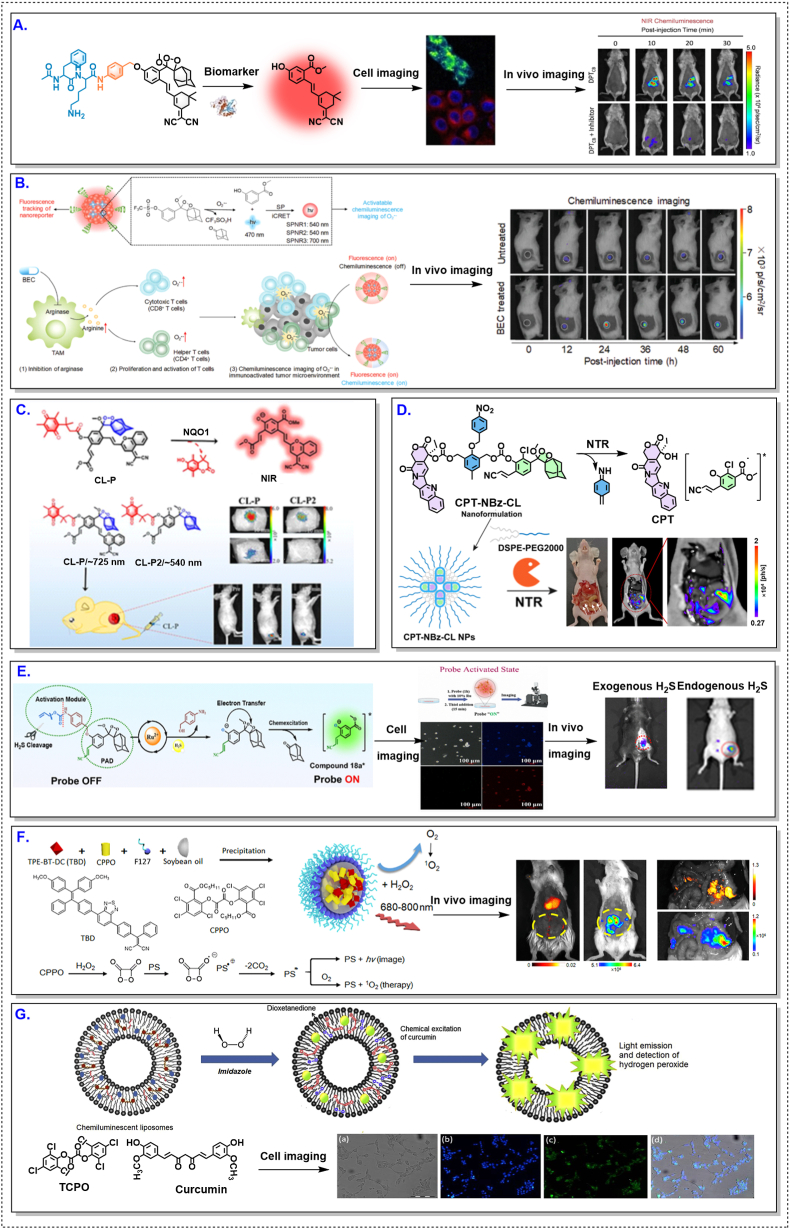
Context-dependent activation of CL probes in breast-cancer models. Representative CL probes evaluated in breast-cancer models, including enzyme-, redox-, immune-, and metabolism-responsive systems. Compared with HCC, signal activation frequently exhibits spatial heterogeneity and variable intensity. These patterns highlight constraints imposed by enzyme accessibility, redox variability, and probe delivery. All above figures were reproduced with proper copyright obtained.

Representative breast-cancer studies [52,65,76,90,101,[105], [106], [107]] illustrating heterogeneous activation and accessibility-limited performance are summarized in Fig. 4 and Table S1.

Accordingly, enzyme-activated designs should be evaluated under realistic delivery conditions, rather than being judged solely on in vitro selectivity or catalytic turnover.

### 4.4 Hypoxia- and pH-responsive probes: conceptual appeal and practical fragility

**Fig. 5:**
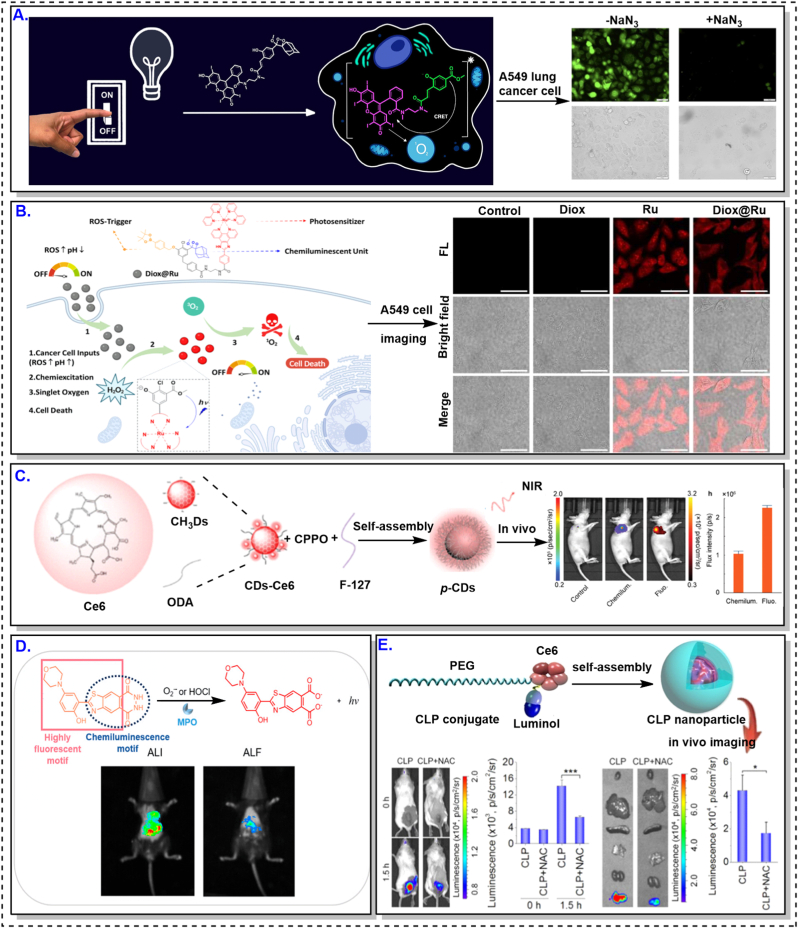
Hypoxia- and inflammation-associated CL imaging in lung-related tumor models. Signal activation is often confined to localized tumor subregions. These examples underscore the fragility of hypoxia- and pH-dependent activation under heterogeneous and poorly perfused conditions. All above figures were reproduced with proper copyright obtained.

Hypoxia- and acidity-responsive CL probes leverage hallmark features of aggressive solid tumors and are frequently proposed as broadly applicable imaging strategies [52,65]. However, as illustrated by tumor contexts such as lung cancer in Table S1 and Fig. 5 [51,53,65,99,106], these triggers introduce intrinsic variability into CL imaging systems.

Hypoxic regions are often poorly perfused, directly limiting probe delivery to the very regions intended for activation. Similarly, acidic microdomains tend to be transient and spatially confined, leading to fluctuating activation efficiency and poor reproducibility. As a result, CL signals derived from hypoxia or pH triggers are frequently localized to tumor subregions, complicating quantitative analysis and longitudinal monitoring.

These observations emphasize that high biochemical specificity does not inherently translate into imaging reliability when activation cues are decoupled from effective probe delivery, a limitation that should be explicitly acknowledged in comparative evaluations.

### 4.5 Delivery, biodistribution, and off-target activation as system-level constraints

Across tumor types and probe classes, delivery efficiency consistently emerges as a dominant determinant of CL performance [85,86,[108], [109], [110], [111]]. Even probes with optimized activation kinetics fail to generate detectable signals if tumor accumulation is insufficient. Moreover, off-target activation—particularly for probes responsive to ubiquitous species such as ROS—can erode the intrinsic signal-to-noise advantages of CL imaging [86].

To summarize, delivery efficiency and off-target activation act as system-level constraints that frequently dominate in vivo CL performance, irrespective of molecular photochemical efficiency.

**Fig. 6:**
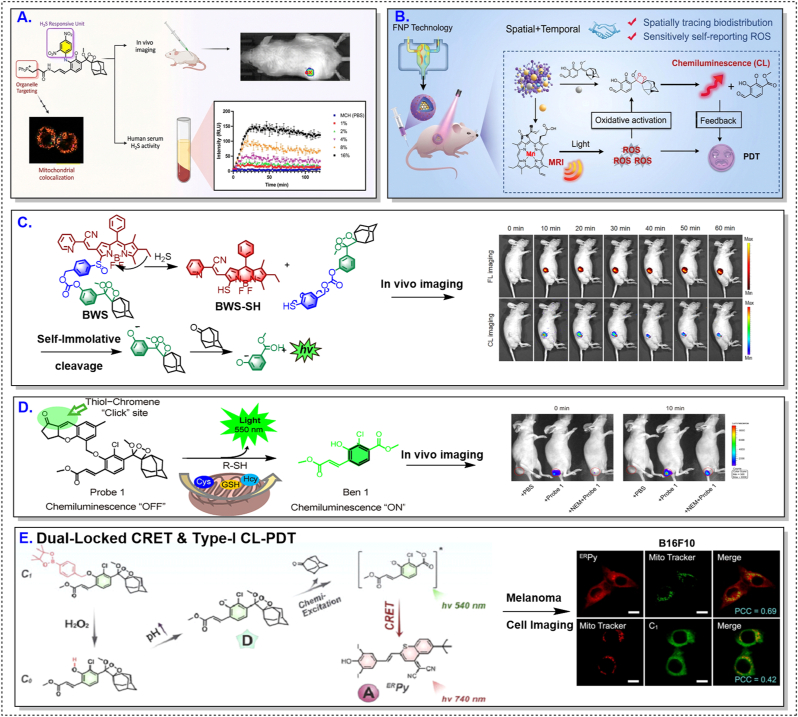
Delivery-dominated performance constraints of CL probes in non-major tumor models. Representative CL probes evaluated in non-major tumor models, including colorectal and oral cancers. Across diverse activation mechanisms, in vivo performance is frequently governed by delivery efficiency and biodistribution. These examples emphasize that CL probes should be assessed as integrated imaging systems rather than isolated molecular designs. All above figures were reproduced with proper copyright obtained.

Together, these examples in Fig. 6 reinforce that delivery and biodistribution frequently dominate in vivo CL performance across non-major tumor models [85,86,[110], [111], [112]]. Failure to account for these factors risks misattributing poor in vivo performance to probe chemistry rather than to biological transport and distribution limitations.

### 4.6 Design implications and limits of generalization

The comparative analysis summarized in Table S1 supports a central conclusion: there are no universal design rules for TME-responsive CL probes. Apparent successes are frequently contingent on tumor models with permissive biochemical environments, while failures arise when probes are applied outside these favorable contexts. Model selection therefore plays a role as critical as molecular design in shaping observed CL performance.

Recognizing these context-dependent constraints provides a necessary foundation for the methodological evaluation frameworks discussed in the following section and motivates a shift toward application-driven probe optimization strategies that explicitly integrate tumor-specific biochemical features with molecular and delivery considerations.

## 5 Methodological considerations and evaluation frameworks for TME-responsive CL imaging

The context-dependent behaviors and failure modes discussed in Section 4 highlight a critical but often overlooked issue in CLI research: many reported performance advantages arise from evaluation practices rather than from intrinsic probe superiority. Without appropriate methodological frameworks, probe brightness, selectivity, and responsiveness can be systematically overestimated, particularly in biochemically permissive tumor models. This section therefore focuses on how CL probes should be evaluated, compared, and interpreted, rather than on what specific probes have been reported.

### 5.1 Why brightness alone is not a reliable performance metric

In CL imaging, photon output is frequently treated as a primary indicator of probe quality. However, unlike excitation-based modalities, CL systems lack external energy input, making observed signal intensity highly sensitive to probe accumulation, activation efficiency, and local biochemical context [57]. As a result, high brightness often reflects favorable tumor microenvironmental conditions rather than superior molecular design [113].

Importantly, brightness measured in a single tumor model does not provide information about robustness, reproducibility, or transferability. Probes exhibiting strong emission in redox-active environments may fail under more restrictive conditions, even when molecular structures are closely related. Consequently, brightness should be interpreted as a conditional outcome rather than an intrinsic probe property [56,114].

### 5.2 Non-comparability across studies: why cross-paper benchmarking is often misleading

Direct comparison of CL probe performance across independent studies is inherently problematic. Differences in tumor models, administration routes, probe dosage, imaging time windows, and detector settings can each alter apparent signal output by orders of magnitude. In addition, tumor stage and intratumoral heterogeneity further confound quantitative comparisons [115,116].

These factors collectively undermine attempts to rank probes based on reported photon counts or signal-to-background ratios alone. Without standardized experimental contexts, such comparisons risk conflating probe chemistry with model-specific or procedural effects. Cross-study benchmarking should therefore be approached with caution unless experimental conditions are explicitly harmonized.

### 5.3 Meaningful optical parameters versus misleading optimization targets

Not all reported optical parameters contribute equally to imaging reliability. Peak intensity, while visually striking, is often dominated by transient activation events and may exaggerate apparent performance. In contrast, parameters such as signal duration, spatial stability, and reproducibility across imaging sessions provide more relevant information for longitudinal or comparative studies [117].

Similarly, emission wavelength shifts toward the near-infrared region can improve tissue penetration, but do not compensate for insufficient probe delivery or unstable activation chemistry [14,118]. These considerations suggest that optimization efforts focused exclusively on peak brightness or spectral tuning risk overlooking more consequential performance determinants.

### 5.4 Complexity penalties in multi-trigger and logic-gated probe designs

Multi-responsive and logic-gated CL probes are frequently proposed as solutions to microenvironmental heterogeneity. While such designs can improve biochemical specificity, they inevitably introduce additional reaction steps that consume reactive intermediates and reduce overall chemiexcitation efficiency.

From a methodological perspective, each added trigger increases sensitivity to pharmacokinetic variability and delivery inefficiencies. In practice, enhanced molecular selectivity may be offset by reduced photon yield and increased signal variability. Complexity should therefore be treated as a design cost that must be justified by demonstrable gains in robustness rather than assumed improvements in selectivity [59,78,118].

### 5.5 The critical role of delivery and pharmacokinetics in performance evaluation

Evaluation frameworks that focus solely on molecular activation chemistry risk misattributing poor imaging outcomes to probe design rather than to delivery limitations. In CL imaging, modest reductions in tumor accumulation can result in disproportionately large signal loss due to the absence of external excitation.

Accordingly, meaningful performance assessment requires explicit consideration of pharmacokinetics, biodistribution, and clearance [56]. Probes that perform similarly at the molecular level may exhibit drastically different imaging behaviors in vivo due to differences in transport and retention [111]. Delivery-aware evaluation is therefore essential for distinguishing chemical limitations from systemic constraints.

### 5.6 Toward standardized evaluation strategies

The issues outlined above point to the need for standardized and context-aware evaluation strategies in CL imaging research. Comparative studies conducted within the same tumor model, using matched delivery protocols and imaging parameters, provide more reliable insights than cross-study comparisons. Likewise, reporting practices that include signal stability, variability, and failure cases would greatly enhance interpretability and reproducibility [119].

Such methodological rigor is particularly important as CL imaging moves toward more complex probe architectures and translational applications. Without it, the field risks perpetuating model-dependent optimization cycles that obscure genuine design principles.

### 5.7 Take-home methodological principles

The following principles emerge from accumulated CL imaging studies:

Brightness is context-dependent and should not be treated as an intrinsic probe metric.
Cross-study performance comparisons are unreliable without standardized conditions.
Robustness, stability, and reproducibility are often more informative than peak intensity.
Increased molecular complexity introduces measurable performance penalties.
Delivery and pharmacokinetics must be integrated into evaluation frameworks.
These principles provide a methodological foundation for interpreting existing studies and for guiding rational design and assessment of next-generation CL probes.

## 6 Outlook and future perspectives

The analysis presented in Section 5 demonstrates that the in vivo performance of CL probes is fundamentally shaped by tumor-type–specific biochemical and physiological features. These constraints arise from intrinsic interactions between chemiexcitation chemistry, tumor microenvironmental heterogeneity, and in vivo transport processes, rather than from isolated shortcomings in probe brightness or reaction kinetics. Similar conclusions have been widely recognized in molecular imaging research, where biological context is considered a primary determinant of imaging performance and translational relevance [[102], [103], [104],^107^]. Future progress in CLI will therefore depend on design strategies that explicitly incorporate tumor microenvironmental conditions rather than relying on generalized optimization.

### 6.1 Embracing tumor-type specificity as a design principle

Accumulating evidence indicates that CL probes rarely exhibit uniform performance across different tumor types. HCC provides a favorable environment for CL activation due to elevated oxidative stress, active sulfur metabolism, and abundant redox- and enzyme-related biochemical pathways. In contrast, breast, colorectal, and other solid tumors frequently display pronounced spatial and temporal heterogeneity in redox status, enzyme expression, and oxygen availability, leading to variable probe activation [107,110].

Attempts to generalize CL probe designs across tumor models often result in reduced signal robustness rather than improved versatility. Accordingly, tumor-type specificity should be regarded as a guiding design principle rather than a limitation. Aligning activation chemistry with dominant microenvironmental features of the target tumor is likely to improve reproducibility and clarify the applicable scope of individual CL probe systems, consistent with broader concepts of microenvironment-driven cancer imaging [[106], [107], [108]].

### 6.2 Balancing multi-trigger complexity with CL efficiency

Heterogeneous activation of single-trigger CL probes has motivated the development of multi-responsive and cascade-activated designs. While such strategies can enhance biochemical selectivity, they also introduce additional reaction steps that may reduce CL efficiency and increase sensitivity to pharmacokinetic variability. Similar trade-offs between selectivity and signal robustness have been noted in both CL and fluorescence-based activatable probe systems [59,76,90].

In practice, increased molecular complexity does not necessarily translate into improved in vivo CL performance. As discussed in Section 5.2, heterogeneous microenvironmental activation in breast and colorectal tumors often leads multi-trigger designs to operate under suboptimal or spatially restricted conditions, where added reaction steps further reduce effective photon output. Multi-trigger designs are therefore most effective when they address specific sources of false activation or false-negative signals, rather than attempting comprehensive biochemical coverage. Given the finite photon budget intrinsic to CL systems, maintaining sufficient chemiexcitation efficiency remains a central design constraint [59,78].

### 6.3 Oxygen availability as an inherent constraint of CL systems

Oxygen dependence represents a fundamental limitation for many CL activation pathways, particularly those based on luminol and peroxyoxalate chemistry. In hypoxic tumors, reduced oxygen availability directly limits chemiexcitation efficiency and contributes to variability in signal output [[81], [82], [83], [84], [85],^110^]. Although alternative strategies, including reductive activation and local oxygen-generating approaches, have been explored, their CL output remains comparatively modest and highly context dependent [58,83].

Rather than attempting to eliminate oxygen sensitivity entirely, future CL probe designs should explicitly align activation mechanisms with tumor oxygenation status. Treating oxygen availability as an inherent constraint, rather than a defect to be universally corrected, is consistent with current understanding of hypoxia biology and its impact on molecular imaging performance [81,85,110].

### 6.4 Delivery and pharmacokinetics as dominant determinants of in vivo CL performance

In CLI, delivery efficiency and pharmacokinetics exert a stronger influence on signal intensity than in excitation-based modalities. Because CL systems cannot compensate for insufficient probe accumulation through external excitation, modest differences in tumor accumulation, clearance, or off-target distribution can lead to disproportionately large variations in imaging outcome [94,111].

Nanotechnology-based delivery strategies have demonstrated potential for addressing delivery-related constraints identified in Section 5.4, particularly in tumor models with limited penetration or rapid clearance [120]. A comprehensive and detailed discussion of CL probes integrated with nanotechnology platforms—including nanoparticle carriers, polymeric assemblies, and hybrid delivery systems—has already been provided in several recent dedicated reviews [54,[121], [122], [123]]. However, despite encouraging progress, many nanoplatforms have not yet achieved consistent advantages over optimized small-molecule CL probes in terms of brightness, reproducibility, or translational feasibility. These observations mirror broader challenges in nanomedicine delivery to solid tumors, where biological barriers often limit effective accumulation despite advanced carrier design [91,106,111].

### 6.5 Toward more systematic evaluation and reporting standards

As the field continues to mature, methodological standardization will become increasingly important. Comparative evaluation of CL probes across multiple tumor models, consistent reporting of optical parameters and imaging conditions, and transparent discussion of limitations are essential for establishing generalizable design principles. Similar needs for standardization and rigor have been emphasized across molecular imaging disciplines to improve reproducibility and cross-study interpretability [[102], [103], [104], [105]].

### 6.6 Summary

CL probe research is entering a stage in which continued progress will be driven not by isolated molecular optimization, but by the systematic integration of chemical design with biological context and performance-oriented evaluation. Importantly, chemical innovation remains the foundation of this evolution: advances in scaffold engineering, activation chemistry, and emission control continue to expand the functional scope and applicability of CL probes across diverse TMEs.

Building on this chemical foundation, complementary strategies such as nanotechnology-based platforms and data-driven methodologies are expected to further amplify the impact of CL probes. Nanoplatforms provide opportunities to translate refined molecular designs into more controllable in vivo systems, enabling spatial regulation, prolonged signal persistence, and multifunctional integration. In parallel, truly computation- or AI-driven optimization of chemiexcitation efficiency and molecular scaffold design remains relatively limited. Existing theoretical and computational studies have primarily focused on elucidating reaction mechanisms, energy-transfer pathways, and structure–efficiency relationships, thereby providing valuable mechanistic insight rather than predictive or generative design frameworks [6,124]. Rather than replacing chemical design, such approaches are more likely to accelerate iterative optimization and experimental triage in design spaces that are difficult to explore empirically.

In summary, the future of CL imaging lies in chemistry-centered, system-aware innovation, where molecular probes, delivery engineering, and computational guidance are developed in concert. This integrated trajectory not only supports deeper mechanistic interrogation of tumor microenvironmental chemistry, but also establishes a scalable pathway toward next-generation CL probes with enhanced robustness, adaptability, and translational relevance.

## CRediT authorship contribution statement

**Dongnan Guo:** Writing – original draft, Conceptualization. **Xiaofang Hou:** Writing – review & editing, Funding acquisition, Conceptualization. **Sicen Wang:** Writing – review & editing, Project administration, Funding acquisition, Conceptualization.

## Ethics approval

Not applicable.

## Declaration of generative AI in scientific writing

Chatgpt was used to correct grammatical errors.

## Funding

The authors are thankful to the 10.13039/501100001809National Natural Science Foundation of China (82473882 82273891).

## Declaration of interests

The authors declare that they have no known competing financial interests or personal relationships that could have appeared to influence the work reported in this paper. All the authors have read and approved the final version of this manuscript.
