# Supplementary material for: Recent advances in chemiluminescence probes for Tumor microenvironment with applications in cancer diagnosis and therapy
Source: Pharm Sci Adv. 2026 Feb 16;4:100113. doi: 10.1016/j.pscia.2026.100113 (PMC12993154; doi:10.1016/j.pscia.2026.100113)
Supplement: Multimedia component 1 [file mmc1.docx]

**Table S1: Representative TME-responsive CL probes and their in vivo performance characteristics**

| **Target** | **CL System** | **Application Model** | **Emission wavelength (nm)** | **CL half-life (min)** | **CL photo yield (%)** | **LOD** | **S/N ratio** | **Penetration depth (mm)** | **Reference** |
| --- | --- | --- | --- | --- | --- | --- | --- | --- | --- |
| ALP | Dioxetane + Porphyrin (CRET) | SMCC-7721 cells;  BALB/c nude mice bearing SMCC-7721 xenografts | dual peaks | >120 | 42.9 (vs. luminol 1%) | 0 – 1000 U/L (linear) | N.R. | N.R. | [1] |
| APN/CD13 enzyme activation | Acryl–Dioxetane | HepG2 cells; Orthotopic HepG2-tumor mice | 540 | ~10 | N.R. | 0.056 | ~250 (in vivo tumor) | 20 (chicken tissue) | [2] |
| CE enzyme activation | Pocket-tailored Dioxetane (CE-2/CE-3) | Orthotopic HCC / intraoperative navigation | 520 | >600 | 7.4 | N. R | N.R. | 10 | [3] |
| FAPα enzyme activation | Adamantylidene–Dioxetane series (CFCL) | HepG2 and LO2 cells; BALB/c nude mice (HepG2 xenografts) | 500–600 nm | N.R. | N.R. | 0.785 ng/mL | N.R. | N.R. | [4] |
| ROS (H₂O₂, O₂⁻) | Bicyclic-dioxetane CP-8 | OVCAR3 xenograft mice | 545 | N.R. | N.R. | 77 mU L⁻¹ | N.R. | N.R. | [5] |
| RNS (ONOO⁻) | Bicyclic Dioxetane nanoprobe (CLNP) | HeLa, HepG2, HT-29, 4T1, HL-60, THP-1, HUVEC, LO2 cells; In vivo: HeLa/HepG2 | 680 | 5 (in vitro) | N.R. | 36 nM | 32–36 fold vs blank | 20 | [6] |
| RNS (ONOO⁻) | Diphenylphosphonate-based CL (CL-1) | HepG2/A549 tumor cells; HepG2-tumor mice; human liver-cancer tissue | 530 nm | N.R. | N.R. | 9.8 nM | 502 | N.R. | [7] |
| H₂O₂-driven + multienzyme-mimetic | Luminol + MOF (MnFe₂O₄ core) | HepG2, HeLa, MCF-7, L02 cells; HepG2 tumor-bearing nude mice | 450 (Luminol); 658/715 (TCPP) | 6 | N.R. | N.R. | N.R. | N.R. | [8] |
| GPC3 antigen detection | Acridinium ester CLIA | In vitro: Clinical serum samples | N.R. | N.R. | N.R. | 0.05 ng/mL | N.R. | N.R. | [9] |
| NTR (hypoxia-related) | Dioxetane–erythrosin B conjugate (CRET-driven) | MCF7, A549, MDA-MB-231 cells | ~540 nm | 10 ± 1 min | 0.02 ± 0.006 % | N.R. | N.R. | N.R. | [10] |
| Cathepsin B (lysosomal protease) | DPT (NIR dioxetane) | 4T1 cells; 4T1 tumor-bearing mice | 650 nm | 14 min | 4.6 × 10^-2^ Einstein /mol | N.R. | 7.6 | N.R. | [11] |
| ENPP-1 (nucleotide metabolic enzyme) | CL-ENPP-1 (Thymidine Phosphodiester dioxetane | MDA-MB-231 cells | green | N.R. | N.R. | ~4500-fold lower LOD than TMP-pNP | ~15000 | N.R. | [12] |
| O₂•⁻ (immune activation readout) | SPNR (semiconducting polymer +encapsulated dioxetane | NDFs, 4T1 cells, CD8+ T cells; 4T1 tumor-bearing mice | 700 nm | ~3 min | N.R. | 11.8 × 10^-9 M | 490-fold (Signal increase) | N.R. | [13] |
| NQO1 (redox enzyme) | CL-P | A549, LO2 cells; A549 tumor-bearing mice | 725 nm | N.R. | N.R. | 0.134 μg/mL | N.R. | 15 | [14] |
| Thiol imbalance / H₂S | BAC Probe (Ru-catalyzed Bioorthogonal Activation Chemiluminescence Probe, A-CN-Ph-O2) | EGC, SKBR-3 cells; Healthy mice, Breast cancer mice | 400–800 nm (broad) | 18.5 h | N.R. | 0.243 µM | 254-fold (vs control) | N.R. | [15] |
| NTR (hypoxia) → prodrug release | CPT-NBz-CL (Theranostic Chemiluminescent Prodrug) | 4T1, HeLa cells; 4T1-Luc1 peritoneal metastasis mouse model) | ~520 nm (Green) | ~1.67 h | N.R. | 0.66 μg/mL | ~19-fold | N.R. | [16] |
| H₂O₂ / MPO (inflammatory phenotype) | CLP Nanoparticles (Self-assembled Ce6-Luminol-PEG conjugate) | 4T1, A549, HCT116; 4T1 tumor-bearing mice | ~440 nm (luminol), ~660–740 nm (Ce6 via chemiluminescence resonance energy transfer, CRET) | N.R. | N.R. | N.R. | N.R. | N.R. | [17] |
| ROS/MPO cascade (inflammatory phenotype) | CLP Nanoparticles (Ce6-Luminol-PEG conjugate) | Neutrophils, A549 cells; Mouse models of peritonitis, liver injury, colitis, and A549 xenografts) | 675 nm | N.R. | N.R. | N.R. | N.R. | >5 (tissue threshold) | [18] |
| H₂O₂ (oxidative stress–driven chemiexcitation) | C-TBD NPs (CPPO and TBD co-encapsulated by pluronic F-127 and soybean oil) | 4T1 cells, 4T1 breast-tumor-bearing mouse, subcutaneous and intraperitoneal metastasis | 600–800 nm (Peak ~660 nm) | 2.3 hr | N.R. | 2 nM | N.R. | N.R. | [19] |
| Diox@Ru molecular platform | Diox@Ru (Schaap dioxetane–Ru(II) conjugate) | 2D monolayer & 3D spheroid CL-PDT | ~608 nm | >720 | N.R. | 25 μM (Activation threshold for H2O2*H*2​*O*2​) | N.R. | N.R. | [20] |
| Dioxetane–Erythrosin B conjugate | CL-E1 (Dioxetane-Erythrosin B Conjugate) | In vitro cell culture | ~ **500–520 nm** | 10 ± 1 min | 0.02 ± 0.006% | N.R. | N.R. | N.R. | [10] |
| CL-P (NQO1-activated NIR probe) | CL-P (NIR dioxetane probe) | A549 and LO2 cells; A549 xenograft tumor model in mice | 725 nm | N.R. | N.R. | 0.134 μg/mL | 4-fold vs visible CL-P2 at 15 mm tissue depth | 15 (chicken breast) | [14] |
| CPT-NBz-CL prodrug nanoparticles | Chemiluminescence (Schaap-type 1,2-dioxetane) | 4T1, HeLa cells; 4T1-Luc1 peritoneal metastasis mouse model) | ~515 nm (Green light) | ~1.67 h (100.2 min) | N.R. | 0.66 μg/mL | 19-fold (vs background) | N.R. | [16] |
| Ce6–Luminol–PEG nanoparticles (CLP) | CLP | 4T1, HCT116, A549 cells; 4T1 tumor-bearing mice | 670 nm (Ce6 emission via CRET, main signal 660–740 nm) | N.R. | N.R. | 10 μM H₂O₂ (linear 10–100 μM) | N.R. | N.R. | [17] |
| p-CDs–Ce6 / Peroxyoxalate nanoplatform | p-CDs = CDs-Ce6 + CPPO + F-127 self-assembly | A549 cells; Tumor-bearing mice | 670 nm | ~25 min | 2.264 × 10^-3 einsteins mol^-1 | 0.18 μM (H2O2) | 25.98 dB | N.R. | [21] |
| MCH (Mitochondria-targeted H₂S probe) | MCH (TPP–dioxetane–dinitrophenyl conjugate) | HCT116, SH-SY5Y, human serum, tumor-bearing mice (in vivo) | 520 nm (green, MC-benzoate emission) | N.R. | N.R. | 0.0028 mM (2.8 µM) H₂S (S/N=3) | N.R. | N.R. | [22] |
| BWS dual-mode probe | BWS (BODIPY-based 1,2-dioxetane conjugate) | HCT116 cells, Tumor-bearing mice | 706 nm (NIR fluorescence) | N.R. | N.R. | N.R. | N.R. | N.R. | [23] |
| Chromene–thiol click-activated CL probe | Probe 1 (Chromene-Adamantane-dioxetane) | HepG2, Hela, SJSA-1, MG-63, HMNNG, SKOV3; Hela, HepG2 Tumor-bearing mice, Human osteosarcoma tissue | 550 nm (yellow-green CL) | N.R. | N.R. | 0.13 µM (Cys), 0.16 µM (Hcy), 0.18 µM (GSH); linear 0–160 µM | N.R. | N.R. | [24] |
| PSA-activated CL probe | CLPSA (Phenoxy-dioxetane-based chemiluminescent probe) | In vitro (PBS), Human semen traces on fabric (Forensic application) | Green photon (N.R) | N. R | N. R | Detection of seminal fluid diluted up to 1:31250 | 157-fold signal-to-background ratio (or 63-fold higher vs fluorescence probe) | N. R | [25] |
| FeDP-Pdots catalytic CL system | FeDP-Pdots-catalyzed L012–H₂O₂ system (CRET) | HeLa, MCF-7, H1299, RAW264.7 cells; HeLa tumor-bearing nude mice) | 470 nm | > 120 min | N.R. | 10 nM (H2O2) | N.R. | N.R. | [26] |
| Pa–Mn&CH–A@P theranostic nanoplatform | Pa-Mn&CH-A@P nanotheranostics (MRI/CL dual-modal) | CAL27, HeLa, A549 cells; CAL27 xenograft tumor-bearing mice | N.R. | N.R. | N.R. | N.R. | 13.5-fold (Pa–Mn&CH-A@P) vs 45-fold (Pa&CH-A@P) CL enhancement | N.R. | [27] |

**References:**

[1] N. Fan, P. Li, C. Wu, X. Wang, Y. Zhou, B. Tang, ALP-Activated Chemiluminescence PDT Nano-Platform for Liver Cancer-Specific Theranostics, ACS Appl Bio Mater 4(2) (2021) 1740-1748. <https://doi.org/10.1021/acsabm.0c01504>.

[2] Y. Liu, J. Zeng, Q. Li, M. Miao, Z. Song, M. Zhao, Q. Miao, M. Gao, An APN‐Activated Chemiluminescent Probe for Image‐Guided Surgery of Malignant Tumors, Advanced Optical Materials 10(14) (2022). <https://doi.org/10.1002/adom.202102709>.

[3] S. Huang, S. Bai, T. Luo, B. Feng, M. Liu, F. Zheng, S. Huang, Y. Fang, D. Ding, W. Zeng, Engineering high-performance chemiluminescent probes via enzymatic pocket targeting: high-throughput screening for ultralong afterglow imaging of orthotopic hepatocellular carcinoma, Science China Chemistry 68(3) (2025) 1175-1184. <https://doi.org/10.1007/s11426-024-2296-6>.

[4] A. Fu, H. Wang, T. Huo, X. Li, W. Fu, R. Huang, Z. Cao, A Novel Chemiluminescence Probe for Sensitive Detection of Fibroblast Activation Protein-Alpha In Vitro and in Living Systems, Anal. Chem. 93(16) (2021) 6501-6507. <https://doi.org/10.1021/acs.analchem.1c00413>.

[5] S.H. Li, G.R. Zhang, Y.T. He, L. Yang, H.L. Li, C.Y. Long, Y. Cui, X.Q. Wang, Emission wavelength-tunable bicyclic dioxetane chemiluminescent probes for precise in vitro and in vivo imaging, Anal. Chem. 95(35) (2023) 13191-13200. <https://doi.org/10.1021/acs.analchem.3c02126>.

[6] M. Shi, Y. Zhang, J.X. Chen, Y. Wu, Z. Wang, P.F. Shi, X. Jin, X.Q. Wang, A Bicyclic Dioxetane Chemiluminescence Nanoprobe for Peroxynitrite Imaging in Vivo, Anal. Chem. 96(48) (2024) 19109-19116. <https://doi.org/10.1021/acs.analchem.4c04510>.

[7] B. Wang, Y. Kong, X. Tian, M. Xu, A highly sensitive and selective chemiluminescent probe for peroxynitrite detection in vitro, in vivo and in human liver cancer tissue, J. Hazard. Mater. 469 (2024) 134094. <https://doi.org/10.1016/j.jhazmat.2024.134094>.

[8] S.Y. Yin, W. Liu, K. Zhang, J. Li, Self-Illuminated, Oxygen-Supplemented Photodynamic Therapy via a Multienzyme-Mimicking Nanoconjugate, ACS Appl Bio Mater 4(4) (2021) 3490-3498. <https://doi.org/10.1021/acsabm.1c00035>.

[9] J.P. Yu, X.G. Xu, R.J. Ma, S.N. Qin, C.R. Wang, X.B. Wang, M. Li, M.S. Li, Q. Ma, W.W. Xu, Development of a clinical chemiluminescent immunoassay for serum GPC3 and simultaneous measurements alone with AFP and CK19 in diagnosis of hepatocellular carcinoma, J. Clin. Lab. Anal. 29(2) (2015) 85-93. <https://doi.org/10.1002/jcla.21733>.

[10] E.M. Digby, M.T. Tung, H.N. Kagalwala, L.S. Ryan, A.R. Lippert, A.A. Beharry, Dark Dynamic Therapy: Photosensitization without Light Excitation Using Chemiluminescence Resonance Energy Transfer in a Dioxetane-Erythrosin B Conjugate, ACS Chem. Biol. 17(5) (2022) 1082-1091. <https://doi.org/10.1021/acschembio.1c00925>.

[11] X. Wei, J. Huang, C. Zhang, C. Xu, K. Pu, Y. Zhang, Highly bright near-infrared chemiluminescent probes for cancer imaging and laparotomy, Angew Chem Int Ed Engl 62(8) (2023) e202213791. <https://doi.org/10.1002/anie.202213791>.

[12] O. Shelef, S. Gutkin, M. Nassir, A. Krinsky, R. Satchi-Fainaro, P.S. Baran, D. Shabat, Thymidine Phosphodiester Chemiluminescent Probe for Sensitive and Selective Detection of Ectonucleotide Pyrophosphatase 1, Bioconjug. Chem. 36(2) (2025) 152-159. <https://doi.org/10.1021/acs.bioconjchem.4c00454>.

[13] D. Cui, J. Li, X. Zhao, K. Pu, R. Zhang, Semiconducting Polymer Nanoreporters for Near‐Infrared Chemiluminescence Imaging of Immunoactivation, Adv. Mater. 32(6) (2019) e1906314. <https://doi.org/10.1002/adma.201906314>.

[14] J. Liu, Z. Chen, H. Huo, L. Chen, Y. Wu, X. Zhang, L. Su, Q. Li, J. Song, An Activatable Near‐Infrared Molecular Chemiluminescence Probe for Visualization of NQO1 Activity In Vivo†, Chin. J. Chem . 40(20) (2022) 2400-2406. <https://doi.org/10.1002/cjoc.202200300>.

[15] D. Guo, D. Xu, Y. Wang, Z. Wang, X. Hou, S. Wang, X. Wei, C. Fan, B. Wang, L. Li, H. Song, W. Yang, A smart Ru-locked chemiluminescence probe via bioorthogonal activation for highly selective, real-time and non-invasive in vivo imaging of thiol dysregulation, (2025). <https://doi.org/10.22541/au.174476364.40441173/v1>.

[16] L. Wei, Y. Wu, Y. Zhou, M. Yang, H. Li, W.C. Geng, Z. Yuan, J. Gao, A Nitroreductase-Activated Chemiluminescent Prodrug for Real-Time Monitoring of Camptothecin Release in Peritoneal Metastasis Theranostics, J. Med. Chem. 68(19) (2025) 20506-20518. <https://doi.org/10.1021/acs.jmedchem.5c01674>.

[17] H. An, C. Guo, D. Li, R. Liu, X. Xu, J. Guo, J. Ding, J. Li, W. Chen, J. Zhang, Hydrogen Peroxide-Activatable Nanoparticles for Luminescence Imaging and In Situ Triggerable Photodynamic Therapy of Cancer, ACS Appl Mater Interfaces 12(15) (2020) 17230-17243. <https://doi.org/10.1021/acsami.0c01413>.

[18] X. Xu, H. An, D. Zhang, H. Tao, Y. Dou, X. Li, J. Huang, J. Zhang, A self-illuminating nanoparticle for inflammation imaging and cancer therapy, Sci Adv 5(1) (2019) eaat2953. <https://doi.org/10.1126/sciadv.aat2953>.

[19] D. Mao, W. Wu, S. Ji, C. Chen, F. Hu, D. Kong, D. Ding, B. Liu, Chemiluminescence-Guided Cancer Therapy Using a Chemiexcited Photosensitizer, Chem 3(6 ) (2017) 991-1007. <https://doi.org/10.1016/j.chempr.2017.10.002>

[20] W.W. Peng, T.J. Zhou, L.F. Hu, V. Vankann, T. Bohn, T. Bopp, S.L. Kuan, T. Weil, Autonomous activation of a gated chemiluminescent photosensitizer enables targeted photodynamic therapy in tumor cells, J. Am. Chem. Soc. 147(31) (2025) 27822-27834. <https://doi.org/10.1021/jacs.5c06761>.

[21] R.W. Song, T.C. Jiang, X.Y. Zhang, C.L. Shen, Q. Lou, C.X. Shan, Triplet Electron Exchange in Carbon Nanodots-assisted Long-persistent near-infrared Chemiluminescence for Oncology Synergistic Imaging and Therapy, Adv Sci (Weinh) 12(5) (2025) e2411898. <https://doi.org/10.1002/advs.202411898>.

[22] H. Gunduz, T. Almammadov, M. Dirak, A. Acari, B. Bozkurt, S. Kolemen, A mitochondria-targeted chemiluminescent probe for detection of hydrogen sulfide in cancer cells, human serum and in vivo, RSC Chem Biol 4(9) (2023) 675-684. <https://doi.org/10.1039/d3cb00070b>.

[23] X.M. Dong, L.X. Sun, Z.W. Zhang, T.L. Zhu, J. Sun, J.Z. Gao, C.J. Dong, R.C. Wang, X.F. Gu, C.C. Zhao, A dual-modality hydrogen sulfide-specific probe integrating chemiluminescence with NIR fluorescence for targeted cancer imaging, Science China-Chemistry 66(6) (2023) 1869-1876. <https://doi.org/10.1007/s11426-023-1579-y>.

[24] B. Wang, X. Tian, X.C. Li, K. Cheng, M. Xu, Thiol-Chromene "Click" Reaction-Activated Chemiluminescent Probe for Thiol Detection In Vitro and In Vivo, ACS Appl Mater Interfaces 15(29) (2023) 34505-34512. <https://doi.org/10.1021/acsami.3c05152>.

[25] S. Gutkin, O. Green, G. Raviv, D. Shabat, O. Portnoy, Powerful Chemiluminescence Probe for Rapid Detection of Prostate Specific Antigen Proteolytic Activity: Forensic Identification of Human Semen, Bioconjugate Chemistry 31(11) (2020) 2488-2493. <https://doi.org/10.1021/acs.bioconjchem.0c00500>.

[26] Y. Teng, M. Li, X. Huang, J. Ren, Singlet Oxygen Generation in Ferriporphyrin-Polymer Dots Catalyzed Chemiluminescence System for Cancer Therapy, ACS Appl Bio Mater 3(8) (2020) 5020-5029. <https://doi.org/10.1021/acsabm.0c00522>.

[27] Y.N. Tao, C.X. Yan, Y. Wu, D. Li, J. Li, Y.C. Xie, Y.S. Cheng, Y.S. Xu, K. Yang, W.H. Zhu, Z.Q. Guo, Uniting Dual-Modal MRI/Chemiluminescence Nanotheranostics: Spatially and Sensitively Self-Reporting Photodynamic Therapy in Oral Cancer, Adv. Funct. Mater. 33(40) (2023). <https://doi.org/10.1002/adfm.202303240>.
